# If it does not help, it might hurt: Pharmacodynamics of a second IVIg course in Guillain–Barré syndrome

**DOI:** 10.1002/acn3.52313

**Published:** 2025-03-14

**Authors:** Sander J. van Tilburg, Ruth Huizinga, Krista Kuitwaard, Sebastiaan D.T. Sassen, Christa Walgaard, Pieter A. van Doorn, Bart C. Jacobs, Birgit C.P Koch

**Affiliations:** ^1^ Department of Immunology, Erasmus MC University Medical Center Rotterdam The Netherlands; ^2^ Department of Neurology, Erasmus MC University Medical Center Rotterdam The Netherlands; ^3^ Department of Neurology Albert Schweitzer Hospital Dordrecht The Netherlands; ^4^ Department of Hospital Pharmacy, Erasmus MC University Medical Center Rotterdam The Netherlands; ^5^ Department of Neurology IJsselland Hospital Capelle aan den IJssel The Netherlands

## Abstract

**Objectives:**

Intravenous immunoglobulin (IVIg) is an effective treatment for Guillain–Barré syndrome (GBS), but recovery varies between patients. This study aims to evaluate the pharmacokinetics (PK) and pharmacodynamics (PD) of a single and a second IVIg dose (SID) in patients with GBS.

**Methods:**

Data were analyzed from the SID‐GBS trial, a double‐blind, randomized, placebo‐controlled study. Patients with poor prognosis (modified Erasmus GBS Outcome Score, mEGOS ≥6) after a standard course of IVIg (0.4 g/kg for 5 days) were randomized to receive either SID or placebo. Serum IgG levels were measured at standard serial time points and clinical outcomes were assessed using the GBS disability score and Medical Research Council sum score. PK modeling was performed to predict IVIg exposure and its association with clinical outcomes.

**Results:**

Serum IgG concentration after a single and double course of IVIg was variable, but accurately described by the current PK model. Lower ΔIgG and IVIg exposure were associated with poorer clinical outcomes. SID increased the IgG concentration, but did not result in an improvement in clinical outcome. Serious adverse events, including thromboembolic events, occurred more frequently in the SID group and were associated with lower IVIg exposure.

**Interpretation:**

SID increases serum IgG levels in GBS patients as predicted by the current PK model, but does not improve clinical outcomes and increases the risk of serious adverse events. Model‐informed precision dosing may guide individualization of treatment.

## Introduction

Guillain–Barré syndrome (GBS) is an acute immune‐mediated polyradiculoneuropathy resulting in a rapidly progressive weakness of the limbs that may extend to cranial nerves and respiratory muscles.^1^, ^2^ Both intravenous immunoglobulin (IVIg) and plasma exchange (PE) are proven effective treatments for patients with GBS,^3^, ^4^ but IVIg is often preferred due to its ease of administration and fewer side effects. Despite treatment, approximately 20% of patients progress during treatment and require respiratory support, about 20% are unable to walk unaided after 6 months, and many experience residual complaints and deficits.^5^ More effective treatments are therefore needed for GBS.

IVIg is a plasma‐derived product pooled from thousands of donors, comprising over 95% IgG. Although patients with GBS receive a standard IVIg course of 2 g/kg body weight over 5 days, they display variable concentrations of serum IgG and clinical responses. Furthermore, a low increase of serum IgG 2 weeks after the start of IVIg treatment was associated with an unfavorable clinical outcome in patients with GBS, which could indicate a dose–response relationship.^6^ Small case studies and series suggested a benefit for serial IVIg courses.^7^, ^8^ In contrast, a recent comparative randomized controlled trial from the Netherlands, the second intravenous immunoglobulin dose (SID)‐GBS trial, showed that a second course of IVIg in patients with a poor prognosis does not improve the outcome on a group level. However, a second course entailed a higher risk for more serious adverse events, including thromboembolic events (TEEs).^9^

Pharmacokinetics and pharmacodynamics (PK/PD) studies can support individualization of drug dosing based on patients' characteristics by identifying covariates and linking dosing to drug concentration and drug effects. The full potential of PK/PD in dosing of drugs in immune‐mediated neuropathies has not yet been used, as only a limited number of models of the PK of IVIg in patients have been described.^10^, ^11^, ^12^ Our recent population PK model for a single course of IVIg in patients with GBS identified concomitant high‐dose corticosteroid therapy and disease severity as covariates for an increased IgG clearance.^11^

In this study, we used data from the SID‐GBS trial and the previously published population PK model to determine the effect of a second IVIg course on serum IgG concentration and clinical outcome. The aim of this study is to investigate the IVIg dose–response relationship and to identify characteristics of patients that potentially benefit from altered dosing regimens, with the ultimate goal of moving toward model‐informed precision dosing.

## Methods

### Study design and data collection

The patients with GBS included in this study participated in the double‐blind, randomized, placebo‐controlled phase 3 SID‐GBS trial (NTR 2224/NL2107).^9^, ^13^ This trial investigated the efficacy of a second IVIg dose (SID; 0.4 g IVIg/kg for 5 days) on patients with a poor prognosis. The modified Erasmus GBS Outcome Score (mEGOS) was assessed 7–9 days after the start of the first standard IVIg course (2 g/kg). Patients with a poor prognosis (mEGOS ≥ 6) were randomly assigned to receive either SID or placebo (Albuman 4%, 8 ml/kg for 5 days). Those with a good prognosis (mEGOS < 6) were not randomized, but they had the same follow‐up and outcome assessments as the randomized patients. All patients fulfilled the Asbury diagnostic criteria for GBS and received the same IVIg regimen of 2 g/kg in 5 days. Patients with a treatment‐related fluctuation (TRF) were excluded from this analysis because another IVIg course is advised in these patients. Serum samples were obtained before the start of treatment, if possible, and 1 week, 2 weeks, 4 weeks, 12 weeks, and 26 weeks after the start of treatment. Total serum IgG concentrations were determined using turbidimetry. The inclusion and exclusion criteria for the SID‐GBS trial have been previously described.^13^

### Clinical data

Patients were evaluated at baseline and at 1, 2, 4, 8, 12, and 26 weeks after the start of the first course of IVIg treatment. The assessments included the GBS disability score (GBS‐DS), which measures mobility and ventilation on a scale from 0 (no disability) to 6 (death), and the Medical Research Council sum score (MRC‐SS) of six bilateral limb muscle groups, which ranges from 0 (quadriplegic) to 60 (normal strength). A TRF was defined as a worsening of more than five points on the MRC‐SS or at least one point on the GBS‐DS following improvement or stabilization of the clinical course for more than 1 week after initial therapy.^14^ Adverse events were documented by the treating physician in accordance with the International Conference on Harmonization Good Clinical Practice guidelines.

### Population PKPD analysis

Previously, we described a two‐compartment population PK model for a single standard IVIg course in patients with GBS.^11^ In this model, we incorporated allometric scaling of body weight on clearance and volume of distribution, a constant endogenous baseline IgG concentration, interindividual variability on clearance and baseline IgG concentration, and increased clearance after concomitant treatment with methylprednisolone and higher disease severity. The model was externally validated using the non‐randomized patients participating in the SID‐GBS trial who had a good prognosis, but the performance for a second IVIg course (randomized patients) was not assessed. In this study, model performance for randomized SID patients was evaluated using visual predictive checks (VPCs), in which the simulated predictions obtained from the population PK model are compared to the observed total IgG concentrations. The total cumulative amount of exogenous IgG in the central compartment (plasma) for each individual patient was simulated using NONMEM (version 7.4.0; ICON Development Solutions, Ellicott City, MD, USA) and PsN (version 4.2.0). The total IgG in the central compartment in a given time window is referred to as exposure throughout this manuscript and essentially represents the area under the curve (AUC) for the concentration‐time profile in a given time window, excluding baseline IgG concentration. Tools used for visual inspection and evaluation of the model were Pirana version 3.0.0 (Certara, Princeton, NJ, USA).

### Statistical analysis

Statistical analysis was performed using R statistical software (version 4.2.2). Time to event analysis was performed using the ‘survival’ package in R and visualized using the package ‘survminer’. *P*‐values less than 0.05 were considered statistically significant.

## Results

### Patient characteristics

Of the 308 patients included in the final SID‐GBS cohort, 238 patients were included in this study (Figure S1). Out of these, 160 patients had a good prognosis after the first standard IVIg course and were not randomized for a second course. From the remaining 78 patients with a poor prognosis, 44 were randomized to receive SID and 34 received placebo 1 week after the first IVIg course. In total, 1028 serum IgG levels were analyzed. The median number of samples for each patient was 4 (range 1–6). Baseline patient characteristics are described in Table 1. Baseline characteristics between included and excluded patients are not different (data not shown).

**Table 1:** Patient baseline characteristics.

	Overall (*n* = 238)	Non‐randomized (*n* = 160)	Placebo (*n* = 34)	SID (*n* = 44)
Demographics				
Age (IQR)	59 (44–68)	54 (41–65)	59 (48–70)	65 (60–74)
Sex (%)				
Male	155 (65)	101 (63)	27 (79)	27 (61)
Female	83 (35)	59 (37)	7 (21)	17 (39)
Clinical features				
Antecedent event (%)				
None	73 (31)	53 (33)	9 (26)	11 (25)
Diarrhea	68 (29)	36 (22)	9 (26)	23 (52)
URTI	57 (24)	38 (24)	12 (35)	7 (16)
Other	40 (17)	33 (21)	4 (12)	3 (7)
NCS subtype (%)				
Demyelinating	136 (58)	86 (55)	24 (71)	26 (59)
Axonal	12 (5)	8 (5)	2 (6)	2 (5)
Equivocal	46 (20)	37 (24)	2 (6)	7 (16)
Inexcitable	11 (5)	0 (0)	4 (12)	7 (16)
Normal	5 (2)	5 (3)	0 (0)	0 (0)
Not performed/N.a.	25 (11)	21 (13)	2 (6)	2 (5)
GBS‐DS entry (IQR)	4 (3–4)	3 (3–4)	4 (4–4)	4 (4–4)
MRC‐SS entry (IQR)	48 (44–52)	50 (47–54)	44 (31–48)	46 (38–49)
mEGOS (IQR)	4 (2–8)	2 (1–4)	10 (8–11)	10 (9–11)
Laboratory				
IgG baseline in g/L (IQR)	11.7 (9.8–13.3)	11.4 (9.4–13.1)	12.5 (11.0–14.2)	12.1 (10.5–13.7)

GBS‐DS, GBS disability score; IQR, interquartile range; IgG, Immunoglobulin G; MRC‐SS, medical research council sum score; N.a., not assessable; NCS, nerve conduction studies; SID, second IVIg dose; URTI, upper respiratory tract infection.

### Dynamics of total IgG in the SID‐GBS cohort

**Figure 1:**
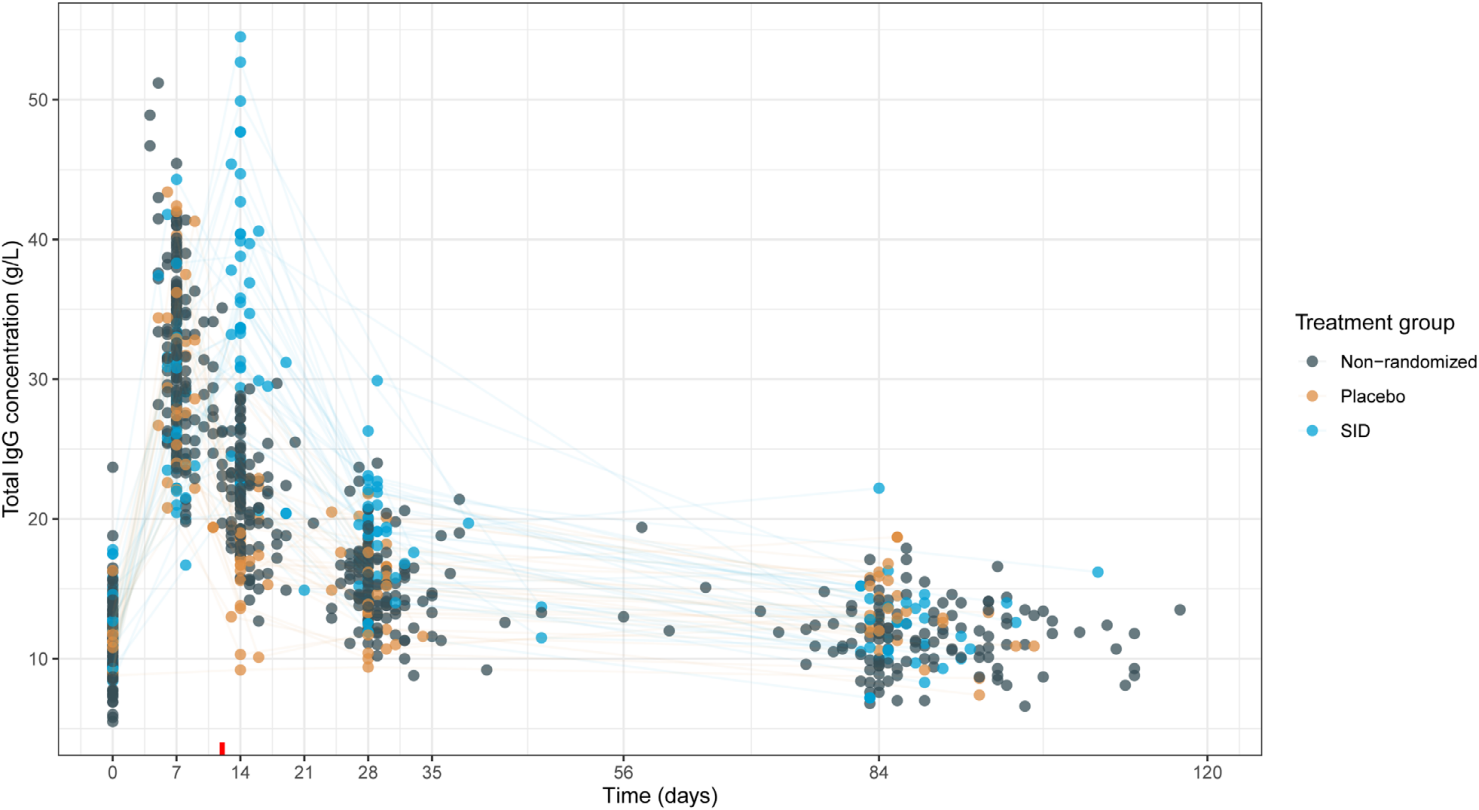
Dynamics of the total IgG concentration in patients stratified by treatment group. The red line on the x‐axis indicates the end of the second IVIg course in the SID group. The colored lines connect observations of individual patients shown only for the randomized patients. IgG, Immunoglobulin G; SID, second IVIg dose.

Figure 1 shows the course of total IgG concentrations in the patients receiving a single IVIg course or SID. Serum IgG levels in all patients increased after the first course of IVIg. Serum IgG concentrations following a SID were elevated for at least 3 weeks compared to those in patients who received a placebo or who had a good prognosis and were therefore not randomized. After randomization, the median IgG concentration for the SID group at week 2 (13–15 days) was 35.5 g/L (IQR = 30.9–42.7) compared with 21.2 g/L (IQR = 18.1–23.2) in the placebo group and 21.8 g/L (IQR = 19.0–23.6) in the group with a good prognosis who had a single course. Similarly, at week 4 (day 26–30), the IgG concentration of 19.4 g/L (IQR = 15.9–21.9) in the SID group was higher than the 15.4 g/L (13.6–17.2) in the placebo group and 15.5 g/L (IQR = 13.8–17.2) in the non‐randomized group.

We subsequently investigated whether the previously developed PK model for IVIg, which was developed based on patients receiving a single standard IVIg course, was also applicable to patients receiving SID. Our analysis showed that the model also adequately predicted the observed total IgG concentrations after SID (Figure S2).

### Observed ΔIgG or exposure is associated with clinical recovery

**Figure 2:**
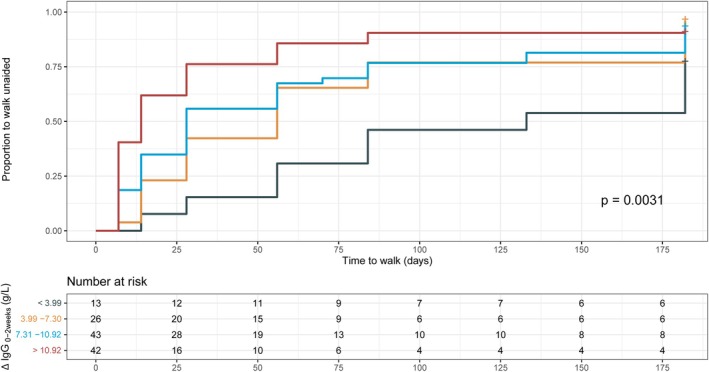
Time to regain the ability to walk unaided in patients with GBS. Patients were stratified into quartiles based on the ΔIgG_0‐2weeks_ as described by Kuitwaard et al.^6^ *P*‐values were derived using the log‐rank test for trend. IgG, immunoglobulin G.

Previously, it was shown that a low increase in total IgG concentration between pretreatment and 2 weeks after treatment was associated with poorer clinical recovery in patients with GBS.^6^ In this independent cohort, patients who received a single IVIg course exhibited a significantly longer time to regain the ability to walk unaided when their ΔIgG_0‐2weeks_ concentrations were lower, using the same cut‐off values as previously described (log‐rank test for trend, *P* = 0.0031; Fig. 2). In the multivariable analysis, patients within the lowest quartile of ΔIgG showed a significantly poorer outcome when adjusted for age, preceding diarrhea, and GBS‐DS at entry (Table S1). However, this association was not statistically significant when adjusted for the MRC‐SS at week 1, a known predictor that is part of the mEGOS.

Similar results were observed when patients were clustered into quartiles of ΔIgG_0‐2weeks_ in this study (cutoff for 25th percentile was 6.7 g/L, for the 50th percentile 9.4 g/L, and for the 75th percentile it was 11.8 g/L, Figure S3). Again, compared to the reference ΔIgG quartile 4, patients in the lowest quartile were less likely to be able to walk unaided (HR: 0.47, 95%CI: 0.27–0.82), but not when adjusted for the MRC‐SS_week1_ instead of the GBS‐DS_entry_ (HR: 1.18, 95%CI: 0.62–2.22; Table S2).

**Figure 3:**
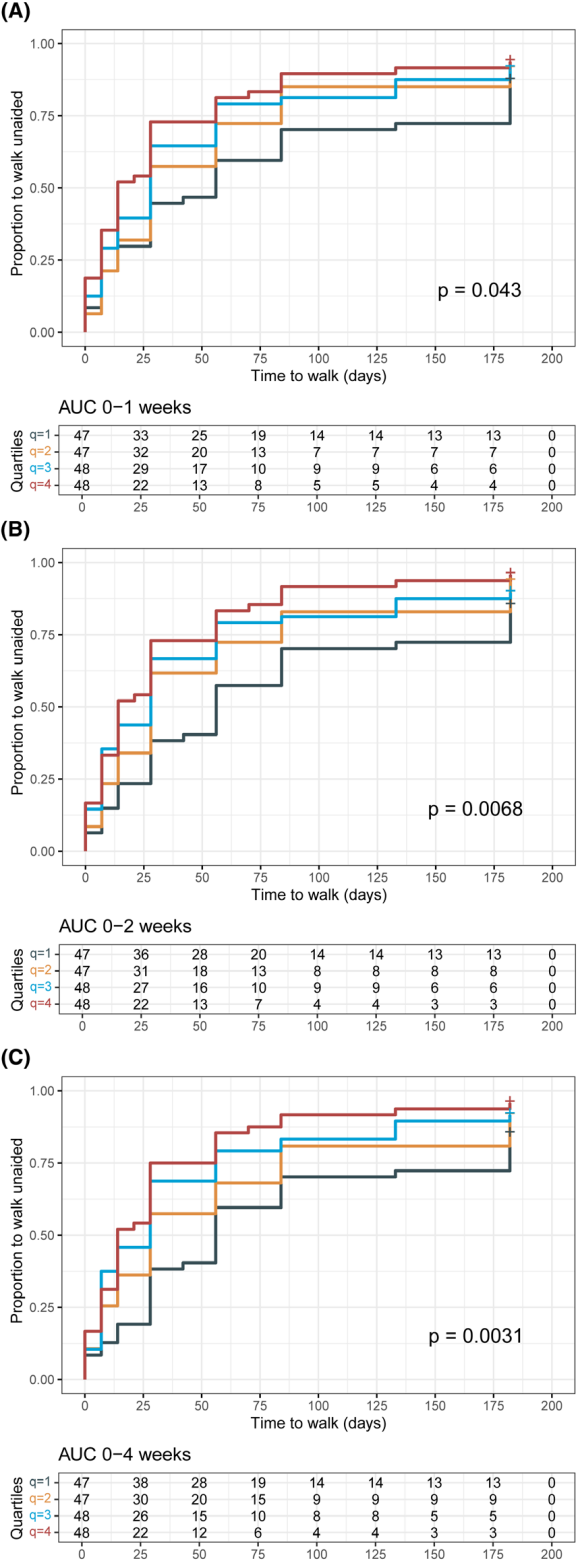
Time to regain ability to walk unaided is prolonged in patients with lower IVIg exposures. Patients were stratified for exposure (AUC) to IVIg between (A) 0–1 weeks, (B) 0–2 weeks, and (C) 0–4 weeks. Only patients who received a single IVIg course are analyzed. *P*‐values were derived using the log‐rank test for trend. AUC, area under the curve; q, quartile.

We then stratified patients into groups based on whether they received a single or second course of IVIg and subsequently clustered according to quartiles of the model‐predicted AUC within a given time window. In patients who received a single IVIg course, significant differences were observed in the time required to regain the ability to walk unaided across quartiles of IgG exposure during the 0–1 week interval (log‐rank test for trend, *P* = 0.043; Fig. 3A), the 0–2 week interval (*P* = 0.0068; Fig. 3B), and the 0–4 week interval (*P* = 0.0031; Fig. 3C). However, IgG exposure had no additional prognostic value when corrected for individual components of the mEGOS for the outcome of regaining the ability to walk unaided (Table S3).

**Figure 4:**
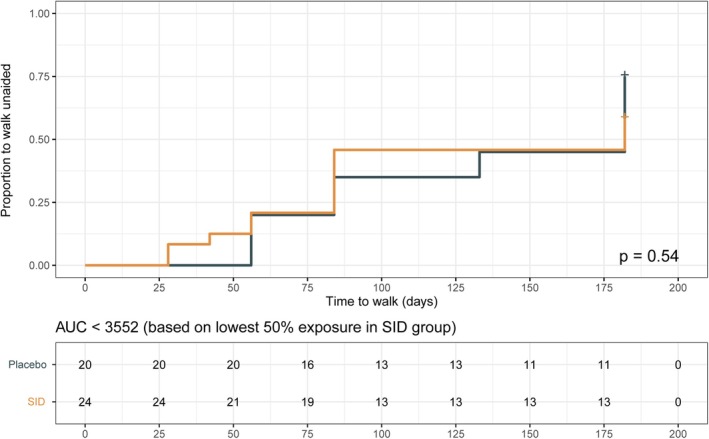
Time to regain ability to walk unaided is similar between patients receiving placebo and SID. Patients were selected based on the 50% lowest AUC in the SID group (AUC < 3552 g·hour/L). This cut‐off was used to filter patients with similar IVIg exposure in the placebo group. *P*‐value was derived using the log‐rank test for trend. AUC, area under the curve; SID, second IVIg dose.

In addition, there is no clear association between clinical outcome and the AUC of IgG between 0 and 1 weeks in the SID group (before the SID; Figure S4). Also, when stratified for the lowest 50% exposure in the SID group, a second IVIg course was not beneficial compared with patients in the placebo group with similarly low exposure (Fig. 4). The IgG exposure had no additional prognostic value to individual components of the mEGOS (Table S4). To assess potential bias, the GBS‐DS was removed as a covariate from the PK model, and similar results were obtained (Figure S5).

### IgG exposure in relation to serious adverse events

**Figure 5:**
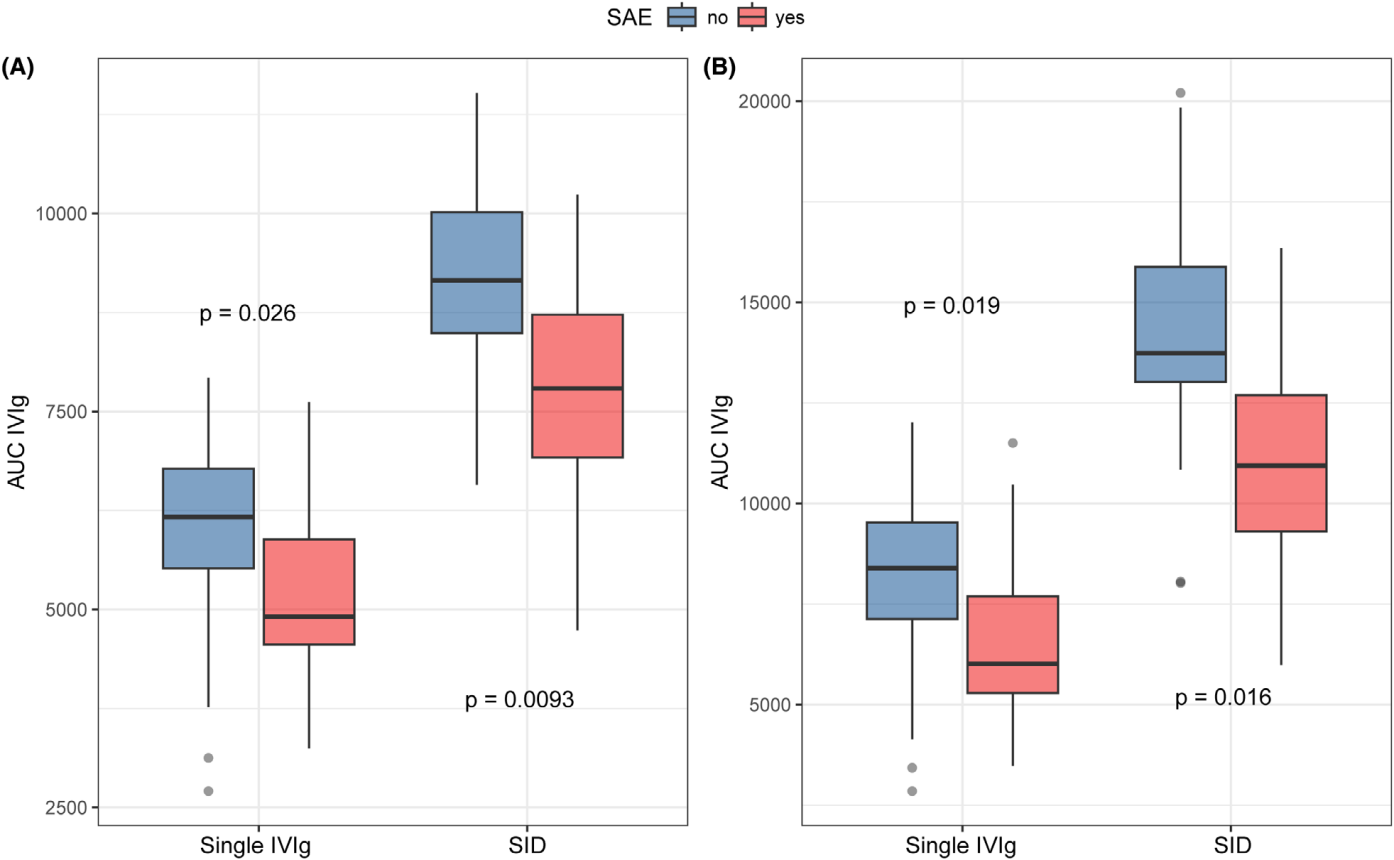
IVIg exposure is lower in patients experiencing serious adverse events. Exposure in the (A) 0–2 weeks and (B) 0–4 weeks interval. Patients were stratified for a single IVIg course or SID. *P*‐values were derived using the Mann–Whitney *U* test. AUC, area under the curve; SAE, serious adverse event; SID, second IVIg dose.

In total, 33 patients experienced serious adverse events in the study cohort, 12/194 (12.8%) in the group receiving a single IVIg course and 21/44 (47.7%) in the SID group. Patients with one or more serious adverse events after IVIg treatment generally had lower IgG exposure during the 0–2 week period compared with patients who did not experience any serious adverse event for both the groups receiving a single IVIg course (Mann–Whitney *U* test, *P* = 0.026) and the group randomized to a SID (Mann–Whitney *U* test, *P* = 0.0093; Fig. 5A). Similar results were observed for the exposure between 0 and 4 weeks (Kruskal‐Wallis test, *P* = 0.019 for single IVIG course, *P* = 0.016 for SID; Fig. 5B).

An additional analysis was conducted in the three patients who developed a serious TEEs after treatment with a SID. In all three patients with TEEs, IgG exposure was significantly lower than in patients without a TEE in the SID group (Kruskal–Wallis test, *P* < 0.05; Figure S6).

## Discussion

In this study, we examined the PK/PD of IVIg treatment in patients with GBS participating in the SID‐GBS trial. Serum IgG levels after the first and second courses of IVIg were highly variable. Patients with the lowest IgG concentrations needed more time to regain the ability to walk unaided. A second course of IVIg increased serum IgG levels, even in patients with low levels after their first course. Despite this increase, patients did not have better outcomes compared to those receiving a single course of IVIg and placebo. Interestingly, patients experiencing serious adverse events after IVIg generally had lower IVIg exposure than those without serious adverse events. The occurrence of TEE seemed to be associated with a low level of IgG rather than higher levels. These results are important for understanding the efficacy of IVIg and the occurrence of side effects in diseases treated with IVIg.

We used a previously described population PK model to predict the exposure of IVIg in blood using simulated individual concentration‐time profiles.^11^ The development of the model was based on patients receiving only a single standard course of IVIg (0.4 g/kg bodyweight for 5 consecutive days). The model was validated in this independent cohort of patients treated with a single course of IVIg. In the current study, we demonstrate that this model also accurately predicts the PK of a second course of IVIg. As expected, after the first 5‐day course, a rapid increase in total IgG levels was followed by a concentration‐dependent decline until the second IVIg course. Generally, after a second dose, an even higher peak level is reached. This population PK modeling approach allows for the correlation of other PK parameters, such as exposure, and is not dependent on the availability of serum samples obtained at standard time points. These results suggest that the previously developed PK model can be utilized to simulate a wide range of different dosing regimens of IVIg in GBS.

We confirmed in an independent cohort of patients our previous findings that low ΔIgG_0‐2weeks_ is associated with worse clinical outcomes in patients with GBS,^6^ and observed a similar association with IVIg exposure and outcomes. In the current study, the quartiles for ΔIgG_0‐2weeks_ levels were <6.7, 6.7–9.4, 9.5–11.8, and >11.8 g/L, whereas in a previous study these were <3.99, 3.99–7.30, 7.31–10.92, and >10.92 g/L.^6^ The discrepancy between quartiles for the ΔIgG_0‐2weeks_ may be explained by differences in disease severity in the cohorts. In previous studies, higher disease severity was identified as a strong predictor of low serum IgG levels. The high rate of consumption of IVIg in these patients may be explained by the high degree of nerve inflammation, similar to the previously observed high rate of IgG consumption in post‐surgical infections.^15^ Other potential mechanisms include increased vascular permeability and extravasation of IgG and a general catabolic state associated with more severe disease. In our previous study, only patients who were unable to walk unaided were included, while in the current cohort, less affected patients could also participate. Here, patients receiving a SID were not included in the calculation of the ΔIgG_0‐2weeks_, so a large proportion of patients with a poor prognosis were not included. Similarly, in a Japanese cohort with fewer patients with severe GBS, a clinically relevant cut‐off was 11.08 g/L for the ΔIgG_0‐2weeks_, where patients below this threshold were significantly less frequently able to walk unaided at 26 weeks.^16^

As low serum IgG levels after IVIg treatment are associated with poor clinical recovery in GBS, we investigated the added value of this biomarker in addition to clinical predictors of the ability to walk independently at 4 weeks and 26 weeks. We confirmed that ΔIgG levels had a significant prognostic value after adjusting for GBS‐DS at admission and for the combined components of the Erasmus GBS Outcome Score (EGOS). The mEGOS is a more accurate and validated prognostic model for GBS, in which the GBS‐DS is replaced by the MRC‐SS at 1 week after admission.^17^ The ΔIgG levels had no additional prognostic value compared to the mEGOS for predicting the ability to walk unaided after 4 and 26 weeks. These results are likely explained by the high association between ΔIgG levels and changes in the MRC‐SS at 1 week. Early prediction is preferred, making a clinical score at entry more useful than at week 1, but the current PK models are not accurate enough to predict individual patients' concentration‐time profiles *a priori*.

Although a second IVIg course (SID) resulted in a further increase in serum IgG concentration, no beneficial effect on outcome was observed in the SID‐GBS trial.^9^ In the trial, randomization was based on poor predicted outcome rather than serum IgG levels, and subgroup analysis showed that patients with low IgG levels at week 1 (the 50% lowest) might favor a second IVIg course. In this study, we showed that patients with the 50% lowest exposures of IVIg between the start of the standard IVIg course and before initiation of the second IVIg course (SID) did not have better clinical outcomes than those with similar exposure randomized to the placebo group. In line with this finding, it was recently shown that the serum neurofilament light chain concentration, a biomarker for axonal damage associated with disease severity in GBS, did not differ over time between the SID and the placebo group, suggesting that an additional IVIg course did not prevent axonal damage.^18^ The recent EAN/PNS 2023 guidelines for GBS also do not recommend a second IVIg course in patients with a poor prognosis.^2^ Our findings support this recommendation, indicating that there is no justification for a second IVIg course 1 week after the start of the first course in patients with severe GBS.

Associations between high‐dose IVIg and increased risk for TEE have been described in literature.^9^, ^19^, ^20^, ^21^ In the SID‐GBS trial, serious adverse events, including three TEE, occurred more frequently in the SID group than in the placebo group.^9^ We observed that patients with serious adverse events generally had lower IgG levels. A potential mechanism for TEE after IVIg is increased plasma viscosity, which results in reduced blood flow.^22^ Two out of three TEE are unlikely to be directly related to IVIg: a radial artery thrombosis after radial arterial line removal and a pulmonary embolism more than 3 months after the last IVIg dose. A recent study in the UK biobank found no increased risk of TEE following IVIg administration in patients without a prior history of such events, but an association with recurrent TEE was observed.^23^ Our findings do not rule out that (extravascular) aggregation of IgG or other mechanisms might have contributed to the occurrence of serious adverse events after high‐dose IVIg courses.

Currently, it is unclear whether low IgG levels are a consequence or a cause of severe GBS. It is possible that administering the SID may occur too late in the disease course to prevent further neurodegeneration. To test this hypothesis, studies with higher initial doses are needed, and adjustments to the dosage should potentially be made during the first IVIg course. Importantly, these adjustments should consider the increased risk of serious adverse events. Additionally, a subset of patients may require a lower IVIg dose, given the limited availability and high cost of IVIg. Multiple studies have shown that dosing strategies using ideal body weight or adjusted body weight are not inferior to current dosing regimens in terms of clinical efficacy and offer cost‐saving opportunities.^24^, ^25^ Novel biomarkers, such as anti‐ganglioside antibody titers,^26^ are crucial for early identification of patients who may benefit from altered dosing regimens. To advance toward model‐informed precision dosing for IVIg, it is essential to identify covariates that explain the variability in the PK of IVIg. Additionally, linking the PK to clinical outcomes is crucial to better identify which individual patients may benefit from altered dosing regimens.

This study has several limitations. First, we did not distinguish between endogenous (pathogenic) IgG and exogenous (therapeutic) IgG (IVIg). In the PK model, we assume a constant endogenous baseline IgG level over the disease course, disregarding potential changes in baseline IgG production or turnover over time. Future studies may distinguish between endogenous and therapeutic IgG based on allotypes.^27^ Second, the group of patients with a poor prognosis receiving SID is relatively low. Third, the model prediction during the first IVIg course (day 0–7) is relatively uncertain given the sparse data in this specific time window. This may lead to inaccurate predictions for the exposure between 0 and 1 weeks and requires validation with more frequent sampling within this period.

In conclusion, our pharmacometric analysis supports previous findings that link the PK of IVIg with clinical outcomes in patients with GBS. We observed no benefit from administering a second dose of IVIg in cases of severe GBS. Model‐informed precision dosing, tailored to individual patient characteristics, has the potential to optimize and personalize IVIg therapy in inflammatory neuropathies, but more biomarkers are needed.

## Author Contributions

ST, BK, and BCJ conceptualized the study. ST, RH, KK, SDS, CW, and PD acquired and analyzed the data. ST, RH, PD, BCJ, and BK drafted the manuscript. All authors read and approved the final version of the manuscript.

## Conflict of Interest

KK reports research grants from Takeda and Grifols (SPIN award) and received speaker's honoraria from CSL Behring and Grifols and a consultancy fee from Takeda, all producers of the product researched. PD reports participation on an advisory board for Octapharma, a producer of IVIg. BCJ reports research grants from Grifols and CSL Behring, which are producers of IVIg. All abovementioned grants and fees were paid to the institution. The other authors declare no relevant conflicts of interest.

## Supporting information


Data S1.

## Data Availability

In compliance with the General Data Protection Regulation, the source data cannot be made available to other researchers as patient approval has not been obtained for sharing coded data. Information about the analytic methods, syntax, and output files of statistical analyses will be made available by the corresponding author upon reasonable request.
