# Supplementary material for: If it does not help, it might hurt: Pharmacodynamics of a second IVIg course in Guillain–Barré syndrome
Source: Ann Clin Transl Neurol. 2025 Mar 14;12(5):966–75. doi: 10.1002/acn3.52313 (PMC12093351; doi:10.1002/acn3.52313)
Supplement: Supplementary file 1 — Data S1. [file ACN3-12-966-s001.docx]

**Supplemental Tables and Figures**

**Contents Page**

Supplementary Figures

Fig S1: Flowchart of the selection of the study cohort 2

Fig S2: Visual predictive check for one and two IVIg courses 3

Fig S3: Time to walk unaided stratified for ΔIgG_0-2week_ 4

Fig S4: Time to walk unaided in SID patients stratified for IVIg exposure 5

Fig S5: Time to walk unaided after removal of GBS-DS from PK model 6

Fig S6: Predicted concentration-time profile for patients receiving SID 7

Supplementary Tables

Table S1: Cox proportional hazard time to walk unaided Kuitwaard et al. 8

Table S2: Cox proportional hazard time to walk unaided this study 9

Table S3: Multivariable logistic regression for patients receiving one IVIg 10

Table S4: Multivariable logistic regression for patients receiving SID 11

References 12

308 included in final analysis SID-GBS trial

112 patients with poor prognosis

99 patients were randomized

215 patients with good prognosis

177 patients eligible for PK analysis

160 patients included

53 allocated
to SID

46 allocated
to placebo

49 SID
patients

17 patients TRF and/or CIDP

7 patients TRF and/or CIDP
3 patients no available IgG measurements

 44 placebo
patients analysed

44 patients included

34 patients included

5 patients TRF
and/or CIDP

**Figure S1.** Flowchart of study cohort selection for patients with GBS. The reasons for excluding patients with a poor prognosis after randomization have been described previously.^1^ The cohort of patients with a good prognosis has been published previously.^2^ Exclusion criteria for this cohort included lack of available serum samples, unknown weight and IVIg dosage, and/or incomplete/insufficient clinical data. *Abbreviations: SID, second intravenous immunoglobulin dose; PK, pharmacokinetic; TRF, treatment related fluctuation; IgG, immunoglobulin G.*


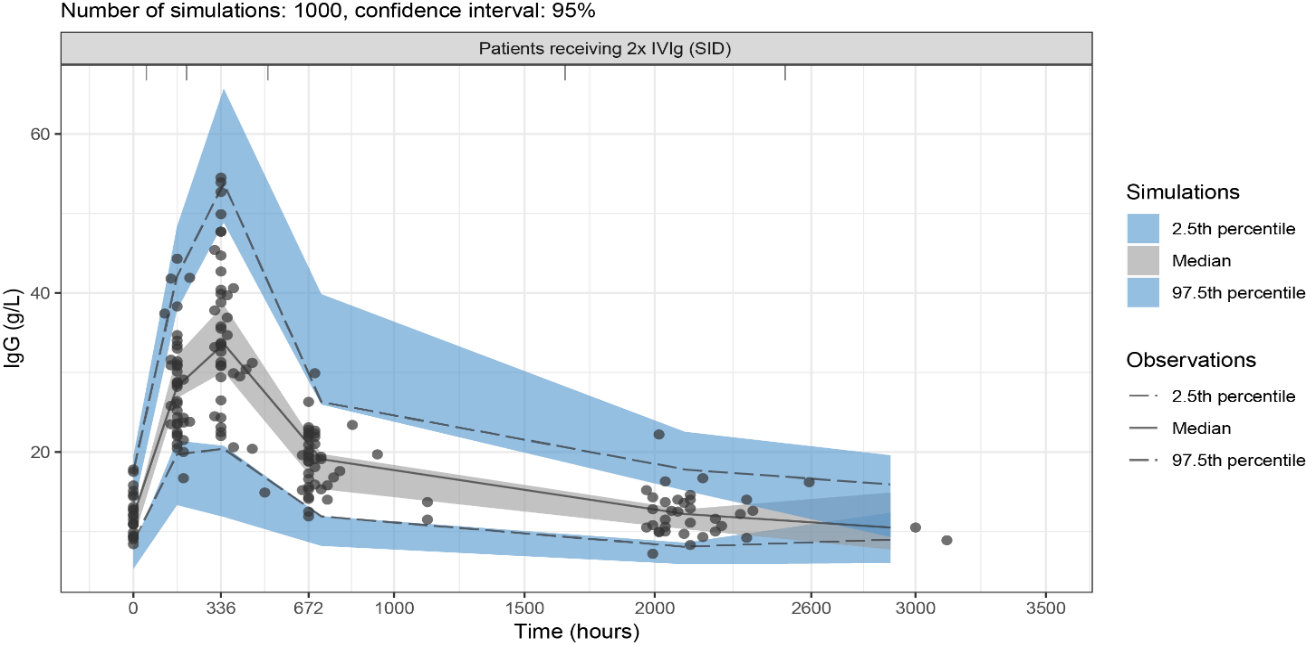

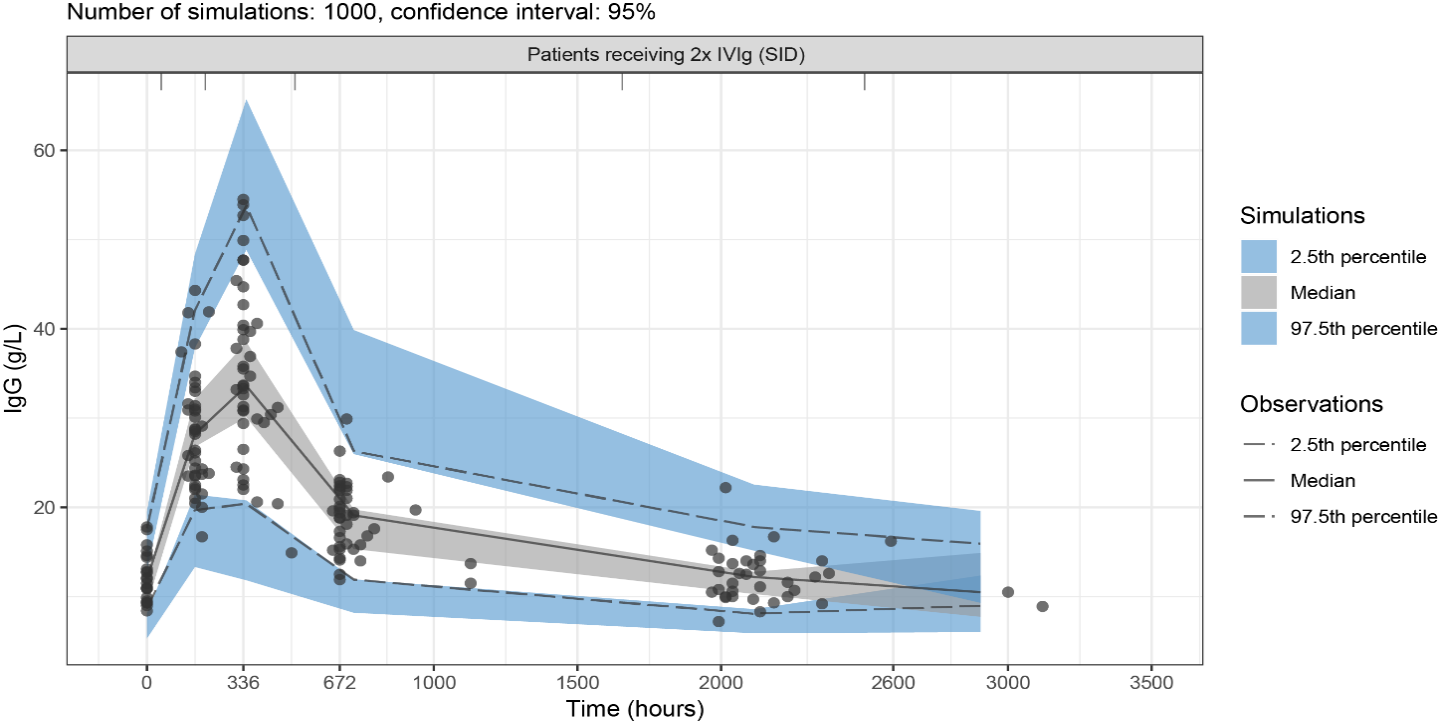


**Figure S2.** Visual predictive check for patients receiving a SID. Final parameter estimates from the previously published population PK model were used (n = 1000 simulations).^2^ The solid line denotes the median of the observations, while the dashed lines represent the 2.5th and 97.5th percentiles of the observed data. The shaded area represents the 95% confidence interval (CI) of the model predicted percentiles. *Abbreviations: SID, second IVIg dose; IgG, Immunoglobulin G.*

**
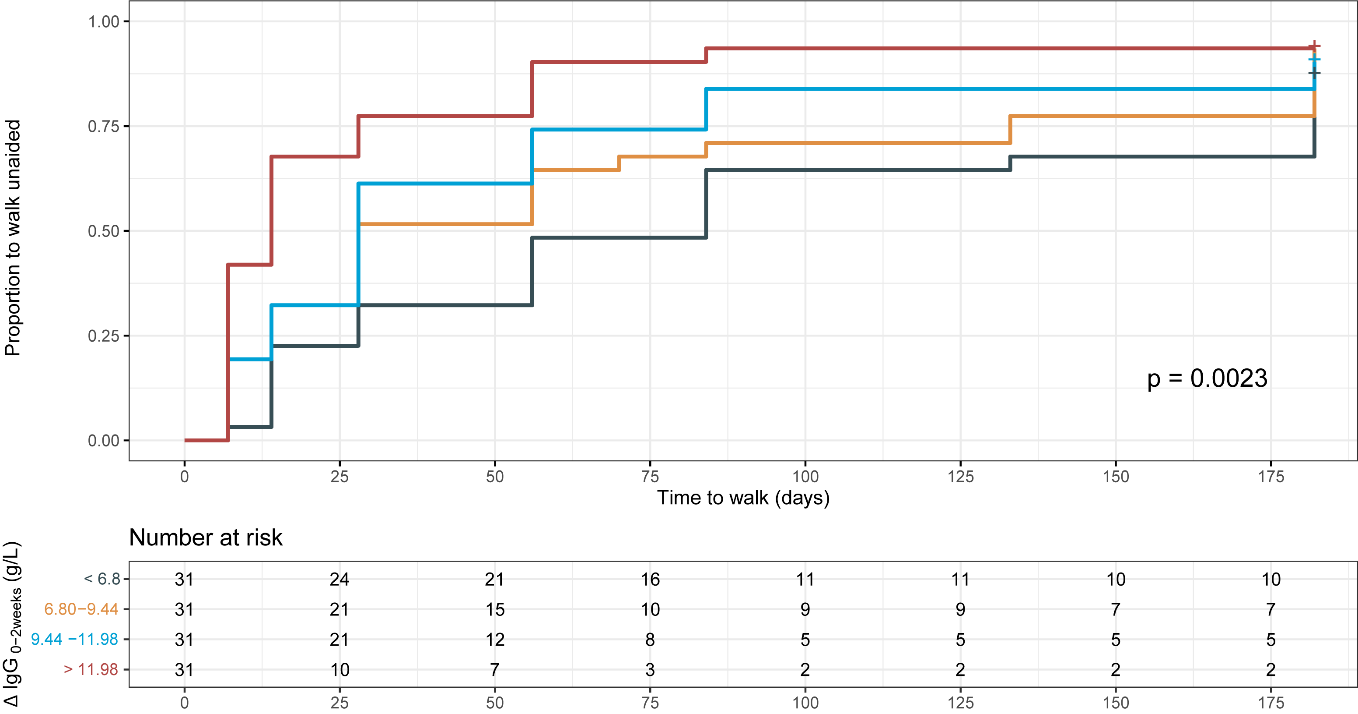
**

**Figure S3.** Time to regain the ability to walk unaided in patients with GBS receiving a single IVIg course. Patients were stratified based on the quartiles ΔIgG_0-2weeks_ as described in this study. P-values were derived using log-rank test for trend. *Abbreviations: IgG, immunoglobulin G.*

**
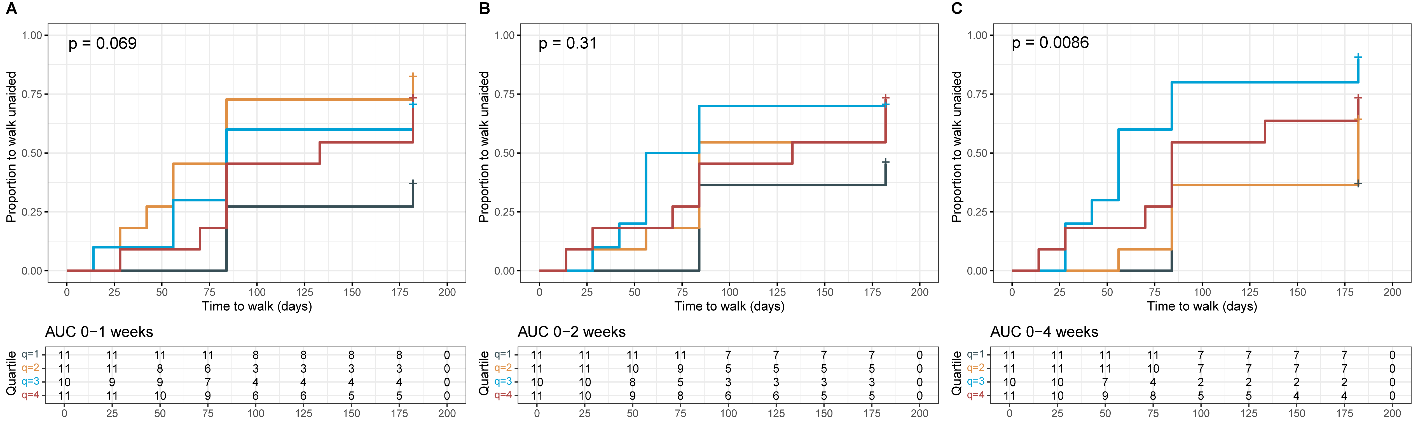
**

**
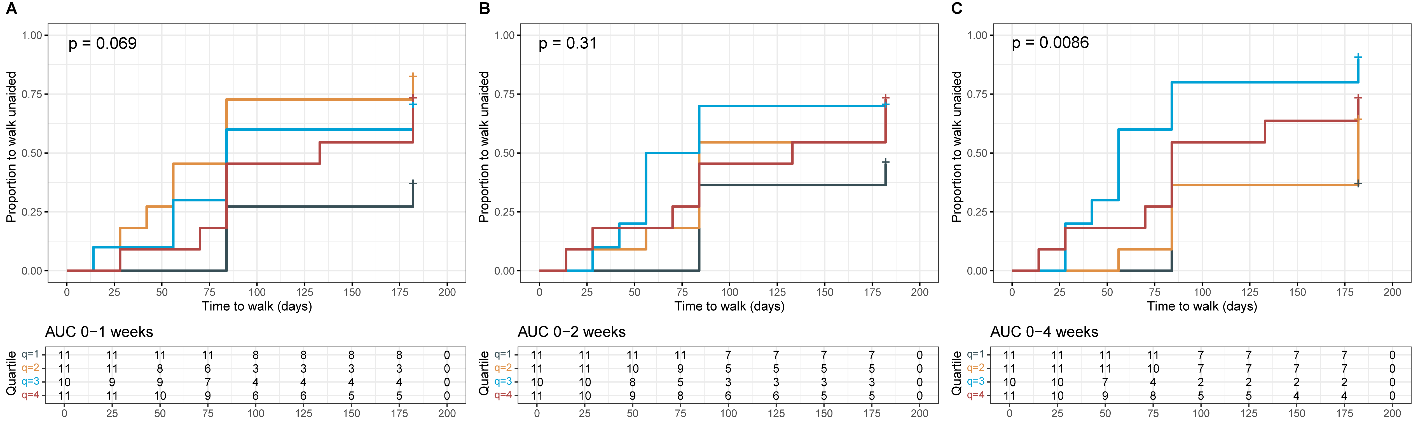
**

**Figure S4.** Time to regain ability to walk unaided in patients receiving SID. Cumulative incidence of the outcome ability to walk unaided was stratified for exposure (AUC) to IVIg between **A)** 0-1 weeks, **B)** 0-2 weeks, and **C)** 0-4 weeks. P-values are derived using log-rank test for trends. *Abbreviations: AUC = area under the curve, q = quartile, SID = second immunoglobulin dose.*

**
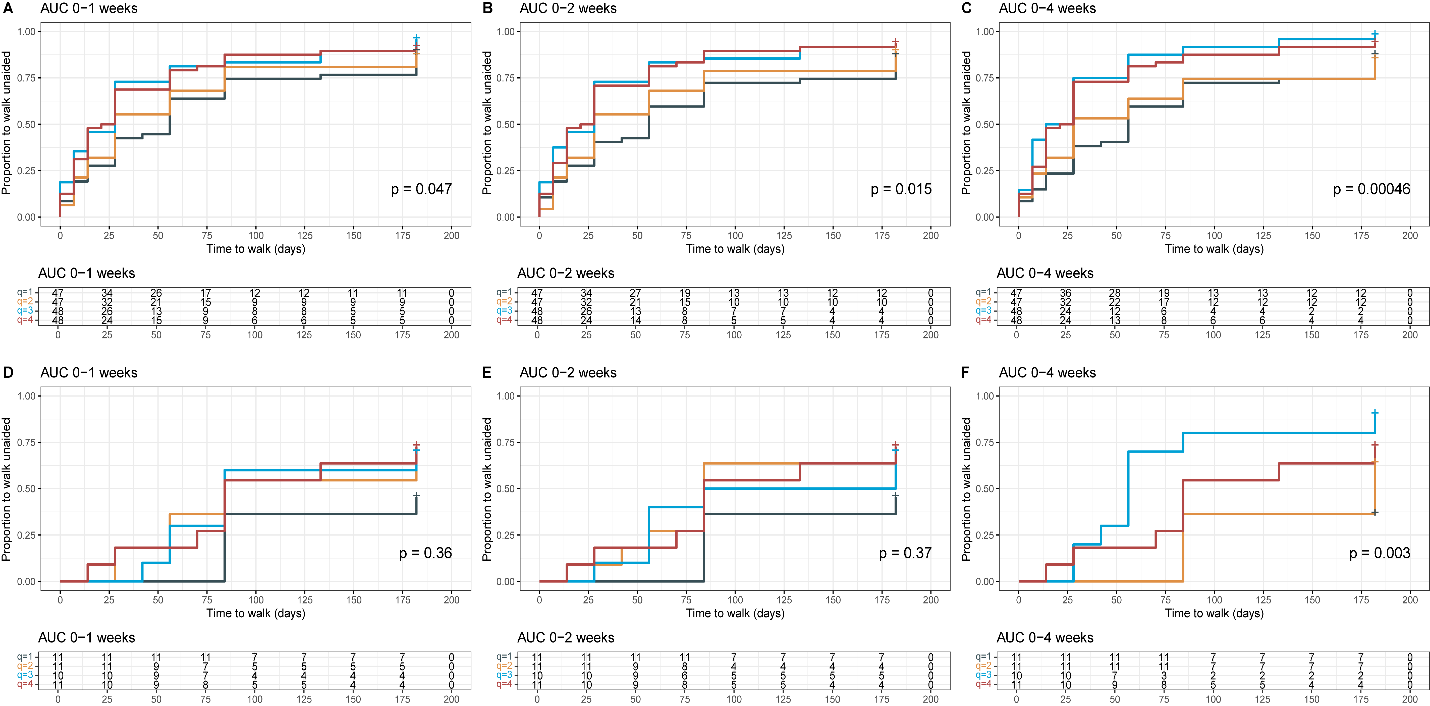
**

**Figure S5.** Time to regain ability to walk unaided. Cumulative incidence of the outcome ability to walk unaided was stratified for exposure (AUC) to IVIg between A) 0-1 weeks, B) 0-2 weeks, and C) 0-4 weeks or patients receiving a single IVIg course or similar but for patients receiving SID (D-F). GBS-DS was removed from the model before simulations. P-values are derived using log-rank test for trends. *Abbreviations: AUC = area under the curve, q = quartile, SID = second immunoglobulin dose.*

**
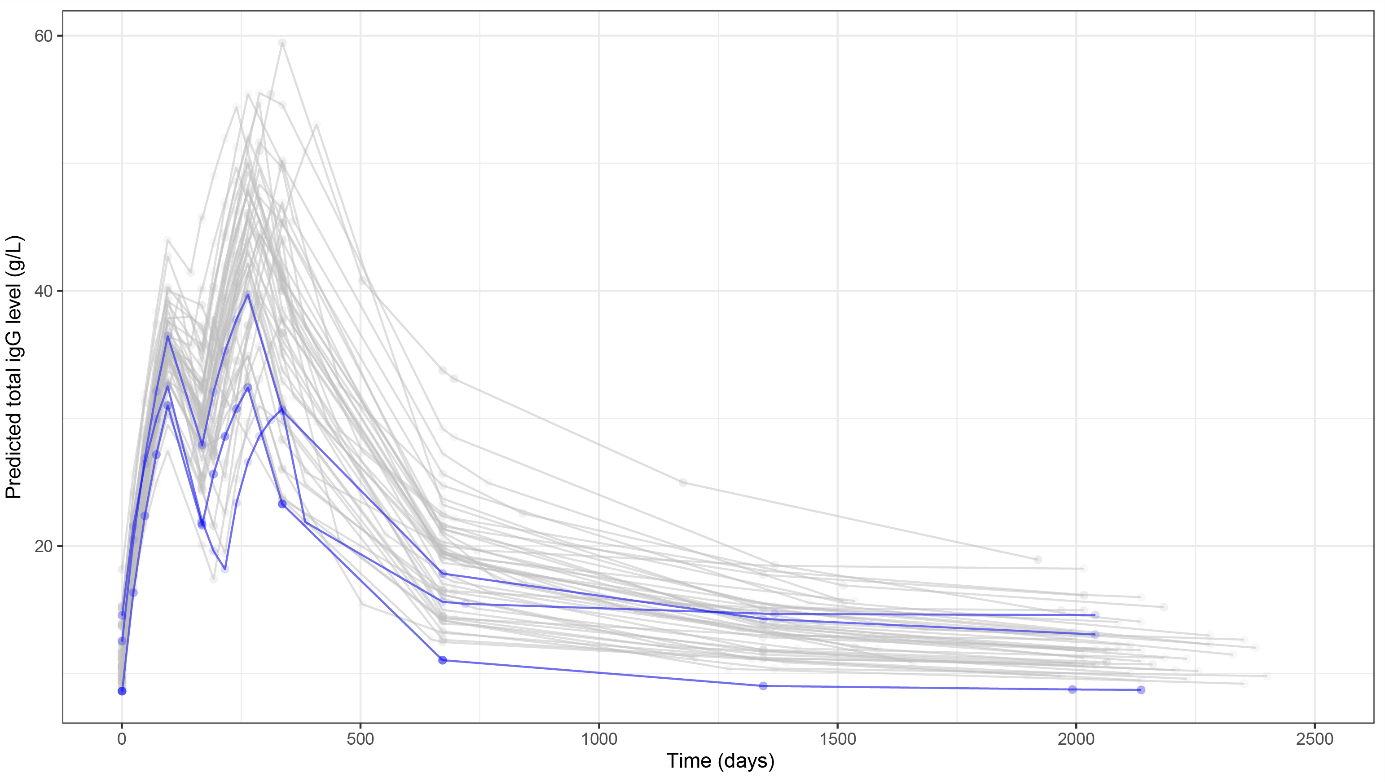
**

**Figure S6:** Predicted concentration-time profile for patients receiving SID. Patients with thromboembolic events are highlighted in blue. Grey lines represent patients without a reported thromboembolic event. *Abbreviations: SID, second IVIg dose; IgG, Immunoglobulin G.*

**Table S1.** Cox proportional hazard for time to walk unaided adjusted for age, preceding diarrhea, and GBS-DS at entry or MRC-SS at week 1. Quartiles are based on Kuitwaard et al.^3^

|  |  |
| --- | --- |
|  | **HR (95% CI)** |
| **Adjusted for GBS-DS at entry** |  |
| Age (years) | 0.99 (0.98-1.00) |
| Delta IgG 0-2 weeks in g/L |  |
| 1st quartile | 0.37 (0.18-0.76) |
| 2nd quartile | 0.69 (0.41-1.15) |
| 3rd quartile | 0.73 (0.46-1.16) |
| 4th quartile | Reference |
| GBS-DS entry |  |
| 3 | Reference |
| 4 | 0.61 (0.42-0.91) |
| 5 | 0.79 (0.47-1.23) |
| Preceding diarrhea | 0.76 (0.47-1.23) |
| **Adjusted for MRC-SS** |  |
| Age (years) | 0.99 (0.98-1.00) |
| Delta IgG 0-2 weeks in g/L |  |
| 1st quartile | 1.07 (0.47-2.41) |
| 2nd quartile | 1.13 (0.66-1.96) |
| 3rd quartile | 0.96 (0.60-1.53) |
| 4th quartile | Reference |
| MRC-SS week 1 | 1.06 (1.04-1.08) |
| Preceding diarrhea | 0.66 (0.41-1.07) |

*Abbreviations: HR, hazard ratio; CI, confidence interval; GBS-DS, GBS disability score; MRC-SS, medical research council sum score; IgG, Immunoglobulin G*

**Table S2.** Cox proportional hazard for time to walk unaided adjusted for age, preceding diarrhea, and GBS-DS at entry or MRC-SS at week 1. Quartiles are based on this study cohort.

|  |  |
| --- | --- |
|  | **HR (95% CI)** |
| **Adjusted for GBS-DS at entry** |  |
| Age (years) | 0.99 (0.98-1.00) |
| Delta IgG 0-2 weeks in g/L |  |
| 1st quartile | 0.46 (0.26-0.81) |
| 2nd quartile | 0.57 (0.33-0.98) |
| 3rd quartile | 0.58 (0.34-1.00) |
| 4th quartile | Reference |
| GBS-DS entry |  |
| 3 | Reference |
| 4 | 0.59 (0.40-0.88) |
| 5 | 0.72 (0.25-2.09) |
| Preceding diarrhea | 0.76 (0.47-1.23) |
| **Adjusted for MRC-SS** |  |
| Age (years) | 0.98 (0.97-0.99) |
| Delta IgG 0-2 weeks in g/L |  |
| 1st quartile | 1.18 (0.62-2.22) |
| 2nd quartile | 1.11 (0.62-1.98) |
| 3rd quartile | 0.84 (0.48-1.46) |
| 4th quartile | Reference |
| MRC-SS week 1 | 1.06 (1.04-1.08) |
| Preceding diarrhea | 0.64 (0.40-1.04) |

*Abbreviations: HR, hazard ratio; CI, confidence interval; GBS-DS, GBS disability score; MRC-SS, medical research council sum score*

**Table S3.** Multivariable logistic regression for patients receiving one IVIg course (non-randomized and placebo groups).

| **Multivariable logistic regression** | **Ability to walk unaided at 4 weeks** | | | |
| --- | --- | --- | --- | --- |
|  | **OR (95% CI)** | **P** | **RR (95% CI)** | **P** |
| IgG AUC week 1* | 0.99 (0.89 – 1.10) | 0.8 | 1.00 (0.97 – 1.02) | 0.75 |
| Age | 0.96 (0.94 – 0.98) | 0.002 | 0.99 (0.98 – 1.00) | 0.0021 |
| Diarrhea | 0.85 (0.36 – 2.04) | 0.7 | 0.99 (0.76 – 1.27) | 0.91 |
| MRC-SS week 1 | 1.17 (1.11 – 1.25) | < 0.001 | 1.06 (1.04 – 1.08) | < 0.001 |
| IgG AUC week 2* | 1.00 (0.96 – 1.05) | 0.8 | 1.00 (0.99 – 1.01) | 0.83 |
| Age | 0.96 (0.94 – 0.99) | 0.003 | 0.99 (0.98 – 1.00) | 0.0025 |
| Diarrhea | 0.85 (0.36 – 2.03) | 0.7 | 0.98 (0.13 – 0.76) | 0.88 |
| MRC-SS week 1 | 1.17 (1.11 – 1.25) | < 0.001 | 1.06 (1.04 – 1.08) | < 0.001 |
| IgG AUC week 4* | 1.01 (0.98 – 1.03) | 0.6 | 1.00 (1.00 – 1.01) | 0.55 |
| Age | 0.96 (0.94 – 0.99) | 0.004 | 0.99 (0.98 – 1.00) | 0.0033 |
| Diarrhea | 0.84 (0.36 – 2.01) | 0.7 | 0.98 (0.76 – 1.26) | 0.86 |
| MRC-SS week 1 | 1.17 (1.11 – 1.24) | < 0.001 | 1.06 (1.04 – 1.08) | < 0.001 |
|  | **Ability to walk unaided at 26 weeks** | | | |
| IgG AUC week 1* | 0.96 (0.83 – 1.12) | 0.6 | 1.00 (0.99 – 1.01) | 1.00 |
| Age | 0.96 (0.92 – 1.00) | 0.049 | 1.00 (0.99 – 1.01) | 0.14 |
| Diarrhea | 0.63 (0.18 – 2.50) | 0.5 | 0.97 (0.88 – 1.07) | 0.54 |
| MRC-SS week 1 | 1.08 (1.05 – 1.12) | < 0.001 | 1.01 (1.00 – 1.02) | 0.0017 |
| IgG AUC week 2* | 0.97 (0.91 – 1.04) | 0.4 | 1.00 (0.99 – 1.00) | 0.81 |
| Age | 0.96 (0.91 – 1.00) | 0.042 | 1.00 (0.99 – 1.00) | 0.13 |
| Diarrhea | 0.63 (0.18 – 2.47) | 0.5 | 0.97 (0.88 – 1.07) | 0.55 |
| MRC-SS week 1 | 1.09 (1.05 – 1.13) | < 0.001 | 1.01 (1.00 – 1.02) | 0.0017 |
| IgG AUC week 4* | 0.98 (0.95 – 1.02) | 0.4 | 1.00 (1.00 – 1.00) | 0.74 |
| Age | 0.96 (0.91 – 1.00) | 0.040 | 1.00 (0.99 – 1.00) | 0.12 |
| Diarrhea | 0.63 (0.18 – 2.50) | 0.5 | 0.97 (0.88 – 1.07) | 0.55 |
| MRC-SS week 1 | 1.09 (1.05 – 1.14) | < 0.001 | 1.01 (1.00 – 1.02) | 0.0016 |

**scaled by factor 1000. OR, odds ratio; RR, risk ratio; AUC, area under the curve (exposure)*

**Table S4.** Multivariable logistic regression for patients receiving SID (two IVIg courses).

| **Multivariable logistic regression** | **Ability to walk unaided at 4 weeks** | | | |
| --- | --- | --- | --- | --- |
|  | **OR (95% CI)** | **P** | **RR (95% CI)** | **P** |
| IgG AUC week 1* | 1.12 (0.77 – 2.11) | 0.6 | 1.11 (0.85 – 1.45) | 0.43 |
| Age | 0.94 (0.84 – 1.04) | 0.2 | 0.95 (0.89 – 1.02) | 0.13 |
| Diarrhea | 2.62 (0.18 – 96.1) | 0.5 | 2.23 (1.57 – 48.71) | 0.61 |
| MRC-SS week 1 | 1.08 (0.99 – 1.24) | 0.2 | 1.07 (0.99 – 1.16) | 0.10 |
| IgG AUC week 2* | 1.12 (0.98 – 1.43) | 0.2 | 1.10 (1.02 – 1.19) | 0.02 |
| Age | 0.95 (0.85 – 1.05) | 0.3 | 0.96 (0.89 – 1.03) | 0.22 |
| Diarrhea | 1.59 (0.04 – 72.5) | 0.8 | 1.38 (0.03 – 54.87) | 0.86 |
| MRC-SS week 1 | 1.10 (0.99 – 1.35) | 0.2 | 1.08 (0.96 – 1.23) | 0.20 |
| IgG AUC week 4* | 1.08 (1.00 – 1.26) | 0.2 | 1.07 (1.00 – 1.14) | 0.03 |
| Age | 0.93 (0.77 – 1.05) | 0.3 | 0.94 (0.84 – 1.05) | 0.29 |
| Diarrhea | 0.95 (0.01 – 63.8) | 0.8 | 0.85 (0 – 204.39) | 0.96 |
| MRC-SS week 1 | 1.15 (1.00 – 1.62) | 0.2 | 1.13 (0.87 – 1.46) | 0.35 |
|  | **Ability to walk unaided at 26 weeks** | | | |
| IgG AUC week 1* | 1.13 (0.92 – 1.42) | 0.2 | 1.05 (0.98 – 1.13) | 0.15 |
| Age | 1.01 (0.95 – 1.08) | 0.7 | 1.00 (0.98 – 1.02) | 0.82 |
| Diarrhea | 0.46 (0.09 – 2.11) | 0.3 | 0.76 (0.50 – 1.17) | 0.21 |
| MRC-SS week 1 | 1.06 (1.01 – 1.12) | 0.018 | 1.02 (1.00 – 1.04) | 0.02 |
| IgG AUC week 2* | 1.03 (0.98 – 1.10) | 0.3 | 1.01 (0.99 – 1.03) | 0.15 |
| Age | 1.01 (0.95 – 1.08) | 0.7 | 1.00 (0.98 – 1.02) | 0.91 |
| Diarrhea | 0.46 (0.09 – 2.10) | 0.3 | 0.75 (0.49 – 1.14) | 0.18 |
| MRC-SS week 1 | 1.06 (1.01 – 1.12) | 0.020 | 1.02 (1.00 – 1.04) | 0.02 |
| IgG AUC week 4* | 1.01 (0.98 – 1.04) | 0.5 | 1.00 (1.00 – 1.01) | 0.37 |
| Age | 1.00 (0.94 – 1.07) | 0.9 | 1.00 (0.98 – 1.02) | 0.96 |
| Diarrhea | 0.46 (0.09 – 2.09) | 0.3 | 0.75 (0.49 – 1.14) | 0.18 |
| MRC-SS week 1 | 1.06 (1.01 – 1.12) | 0.017 | 1.02 (1.00 – 1.04) | 0.02 |

**scaled by factor 1000. OR, odds ratio; RR, risk ratio; AUC, area under the curve (exposure)*

**References**

1. Walgaard C, Jacobs BC, Lingsma HF, et al. Second intravenous immunoglobulin dose in patients with Guillain-Barré syndrome with poor prognosis (SID-GBS): a double-blind, randomised, placebo-controlled trial. *The Lancet Neurology* 2021; **20**(4): 275-83.

2. Fokkink WJR, van Tilburg SJ, de Winter BCM, et al. Population Pharmacokinetic Modelling of Intravenous Immunoglobulin Treatment in Patients with Guillain-Barré Syndrome. *Clin Pharmacokinet* 2022; **61**(9): 1285-96.

3. Kuitwaard K, de Gelder J, Tio-Gillen AP, et al. Pharmacokinetics of intravenous immunoglobulin and outcome in Guillain-Barré syndrome. *Ann Neurol* 2009; **66**(5): 597-603.
